# A Survey on COVID-19 Data Analysis Using AI, IoT, and Social Media

**DOI:** 10.3390/s23125543

**Published:** 2023-06-13

**Authors:** Muhammad Junaid Butt, Ahmad Kamran Malik, Nafees Qamar, Samad Yar, Arif Jamal Malik, Usman Rauf

**Affiliations:** 1 Department of Computer Science, COMSATS University Islamabad (CUI), Islamabad 45550, Pakistan; mjunaid0093@gmail.com (M.J.B.); samadyark0313@gmail.com (S.Y.); 2 School of Health and Behavioral Sciences, Bryant University, Smithfield, RI 02917, USA; nqamar@bryant.edu; 3 Department of Software Engineering, Foundation University, Islamabad 44000, Pakistan; arif.malik@fui.edu.pk; 4 Department of Mathematics and Computer Science, Mercy College, Dobbs Ferry, NY 10522, USA

**Keywords:** COVID-19, IoT, artificial intelligence, data analysis, epidemic outbreak, social network analysis

## Abstract

Coronaviruses are a well-established and deadly group of viruses that cause illness in both humans and animals. The novel type of this virus group, named COVID-19, was firstly reported in December 2019, and, with the passage of time, coronavirus has spread to almost all parts of the world. Coronavirus has been the cause of millions of deaths around the world. Furthermore, many countries are struggling with COVID-19 and have experimented with various kinds of vaccines to eliminate the deadly virus and its variants. This survey deals with COVID-19 data analysis and its impact on human social life. Data analysis and information related to coronavirus can greatly help scientists and governments in controlling the spread and symptoms of the deadly coronavirus. In this survey, we cover many areas of discussion related to COVID-19 data analysis, such as how artificial intelligence, along with machine learning, deep learning, and IoT, have worked together to fight against COVID-19. We also discuss artificial intelligence and IoT techniques used to forecast, detect, and diagnose patients of the novel coronavirus. Moreover, this survey also describes how fake news, doctored results, and conspiracy theories were spread over social media sites, such as Twitter, by applying various social network analysis and sentimental analysis techniques. A comprehensive comparative analysis of existing techniques has also been conducted. In the end, the Discussion section presents different data analysis techniques, provides future directions for research, and suggests general guidelines for handling coronavirus, as well as changing work and life conditions.

## 1. Introduction

In the last week of December 2019, in Wuhan, China, the first patient of COVID-19 was reported [1]. Compared to normal flu and cold, the novel coronavirus is more dangerous for humans and especially for the human immune system. There are two well-known coronavirus diseases, Severe Acute Respiratory Syndrome (SARS) and Middle East Respiratory Syndrome (MERS), which have death rates of 13% and 34%, respectively. As we are writing our survey, COVID-19 has spread to 228 countries and territories. The death toll and number of infected cases have reached 6.89 million and 690.07 million (as of 9 June 2023), respectively, however, it is still being predicted that it can pose an even greater threat to public health [2]. The mortality rate due to COVID-19 is faster than other respiratory-related diseases.

**Figure 1:**
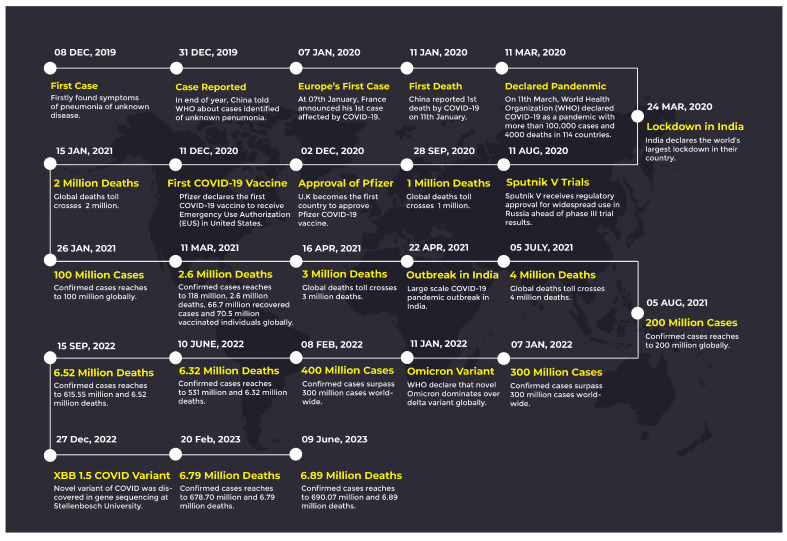
Timeline of COVID-19 pandemic [2].

In the early stages, the Chinese government did not recognize the coronavirus, and they declared that some unknown symptoms relevant to pneumonia are found in a patient due to an unknown disease. On 31 December 2019, the Chinese government told the World Health Organization (WHO) about an unknown disease which many people in China were infected with. On 7 January 2020, the first patient infected by COVID-19 was reported in France. In the same month, on 11 January, the first death due to COVID-19 was reported in China. From here, the journey of COVID-19 started and it rapidly spread all over the world and the number of infected people and death toll grew worldwide. On 11 March, the WHO declared COVID-19 a pandemic and put restrictions on air travel. As we are writing this survey, the number of infected patients and the death toll has reached millions. Figure 1 shows the number of deaths since the start of the pandemic on a timeline.

In the last two years, many research papers and preprints have been published on various platforms to analyze pandemic-related data in order to gain a better understanding of and cure for the disease. These papers covered various aspects such as how Artificial Intelligence (AI), Machine Learning (ML), and the Internet of Things (IoT) play an important role in the global war against the COVID-19 pandemic. Some research articles used Social Network Analysis (SNA) to uncover how misinformation and fake news related to COVID-19 are being spread over the Internet. Some of the papers only covered the topics of digital activism and social media. Looking at the related published survey papers, a few of the survey papers only covered the applications of AI to combat COVID-19 [3]. Some only gathered and discussed various kinds of open-source datasets related to COVID-19 [4]. A few other survey papers covered the forecasting models for coronavirus disease [5] and the impact of COVID-19 on teaching and education in different countries [6]. In this survey, we have tried our best to cover the topics related to COVID-19 and its data analysis, such as AI, ML, blockchain, digital activism, Internet of Things, social media, and SNA. Moreover, we also discuss the impact of COVID-19 and its related activities on human social life and social networks. This paper describes how fake news, altered results, and formed theories were spread over social media, such as Twitter, by applying various SNA and sentimental analysis techniques. Furthermore, we also highlight how activities such as education and office work need to be shifted from a physical to a digital workplace. Data literacy in research refers to having the ability to efficiently navigate and utilize data, ensuring the reliability and validity of research findings. It enables researchers to make decisions based on facts, contribute to the body of existing knowledge, and advance scientific knowledge in their specialized fields. In other words, it is a multifaceted idea that draws interest from numerous communities of practice from various perspectives [7]. The authors divided these communities of practice into four groups, digital activism, artificial intelligence and machine learning, social network analysis, and social media. Most significantly, the predominant body of work focuses on the concept of data literacy within the realm of artificial intelligence and machine learning. Additionally, there exists a significant body of literature that highlights the importance of data literacy within social network analysis and within the realm of social media.

The COVID-19 pandemic revealed several challenges, whether AI is helpful in social control, searching for care and vaccine, COVID-19 diagnoses and prognoses, tracking and forecasting the spreading of the virus? As this paper surveys the novel COVID-19 pandemic covering various research areas and after a brief history of the pandemic, we provide a taxonomy of the related disciplines and their use in coronavirus data analysis. At the end of the paper, a discussion with future directions is presented for each part of the taxonomy.

This survey is focusing on several potential COVID-19 related research areas that played a role directly or indirectly. The major contributions of this survey paper are to present a detailed analysis of COVID-19 and related developments in AI, ML, Deep Learning (DL), and IoT techniques that are currently being developed to assist in managing or combating COVID-19, the application of the Internet of Things (IoT), the in-depth study of mainstream social media (Twitter, Facebook, and Instagram), how social media played a negative role during the pandemic, and the effect of the pandemic on human lives and transformation of physical activities to the digital world. In Section 5, we also discuss the accuracy and dataset size used for the appropriate techniques. Almost every aspect of COVID-19 related research is covered in our survey so that a novice researcher can analyze previous works and find a better solution.

The rest of the work is organized as follows. Section 2 presents the background of pandemics in the previous century, such as the Spanish flu, Asian flu, Hong Kong flu, and SARS. Section 3 describes the research work related to the main components of the taxonomy. Section 4 describes digital activism and social media conspiracies in detail. AI and ML applications are discussed in Section 5 whereas SNA-based studies and issues are provided in Section 6. Section 7 presents a discussion and future directions and Section 8 concludes the paper.

## 2. Background and Motivation

This section presents various pandemics of the last century and the motivation for our research.

### 2.1. Pandemics in the Last Century

The past century has seen an abundance of various epidemic outbreaks. Historically HINI, H2N2, H3N2, and recently SARS-CoV and MERS-CoV, have been declared as a foundation for most of the outbreaks and epidemics in the last 105 years and many are expected in future, as shown in Table 1. The HIN1 was declared a foundation for two pandemics, the Spanish Flu and Swine Flu, which were active in 1918–1919 and 2009–2010, respectively; while other viruses such as H2N2 and H3N2 were declared responsible for introducing Asian Flu and Hong Kong Flu in 1957–1358 and 1968–1969 accordingly. Under this heading, we provide a short overview of some well-known pandemics which resulted in millions of deaths in the last century and detail how many people are still suffering from them.

#### 2.1.1. Spanish Flu Pandemic (1918–1919)

The Spanish Flu emerged in 1918 and is still considered one of the most fatal viruses in the history of humankind. This flu also falls into the category of a pandemic and is caused by the H1N1 virus. At that time, it was declared that the origin of this virus was birds. According to estimated figures, it infected more than 500 million people and caused more than 50 million deaths, including 675,000 Americans. The death toll of this virus was more than the number of deaths in World War I. This flu was first found in Europe, the United States, and some parts of Asia. The majority infected by the Spanish Flu was the young and healthy generation. It attacked its hosts by injecting cytokine into their immune systems which led to death.

#### 2.1.2. Asian Flu Pandemic (1957–1958)

The Asian Flu Pandemic, also known as the 1957 flu pandemic was the second deadliest pandemic of the 20th century and was first triggered in February 1957 in East Asia. After that, it spread very fast to various countries via ships, trains, and aeroplanes. According to raw data, it marked around 116,000 deaths in the United States and a total of 1.1 million approximately worldwide. The H2N2 virus was declared the cause of the Asian Flu pandemic. After 11 years, this virus stopped affecting human hosts due to the occurrence of changes in the human immune system.

#### 2.1.3. Hong Kong Flu Pandemic (1968–1969)

The Hong Kong Flu belongs to the second category of flu; it came into existence on 13 July 1968 and survived till 1969. It was declared the third influenza pandemic of the 20th century which caused, approximately, one million deaths globally. The fundamental cause of this pandemic was the H3N2 virus, which originated from the H2N2 virus that was mainly introduced by the Asian flu pandemic. This virus mainly targeted those people who were above 65, unlike the Spanish flu pandemic.

#### 2.1.4. Severe Acute Respiratory Syndrome (SARS/2002–2004)

It originated from SARS-CoV and was declared a global outbreak from 2002 to 2004. During these two years, this outbreak infected over 8000 people and caused around 774 deaths worldwide. The major causative agent of this virus was SARS-CoV which was mainly inherited from bats. Later on, it was proved that SARS-CoV was shifted from bats to other animals and then transferred to humans.

#### 2.1.5. COVID-19 (2019–Present)

COVID-19 refers to the coronavirus disease that first appeared in 2019. This virus is considered a deadly virus that has a resemblance to previous viruses. It officially came into existence in late December 2019 when China reported a few cases of an unknown infection to the WHO that presented similar symptoms to pneumonia. Later on, this deadly virus spread rapidly across the globe and urged the whole world to take measures to restrict it. There are various theories based on the cause of COVID-19. Some people consider it an international conspiracy against humans. However, it still exists, claims thousands of lives and millions of patients are infected by this deadly virus every day.

### 2.2. Comparison of Existing Research

In the last couple of years, extensive research has been completed regarding COVID-19. In addition, many survey papers have been written to highlight the importance of the application of AI, ML, DL, blockchain, Internet of Things, etc. However, each survey paper covered a specific area of research. A few research papers only covered the application of AI, whereas some other authors focused on ML, DL, or the application of blockchain. With this in mind, this study covers almost every field of research regarding COVID-19, such as applications of AI, ML, DL, blockchain, Internet of Things, SNA, impact of social media, and digital activism. In Table 2, we compared existing surveys and studies with our survey paper.

**Figure 2:**
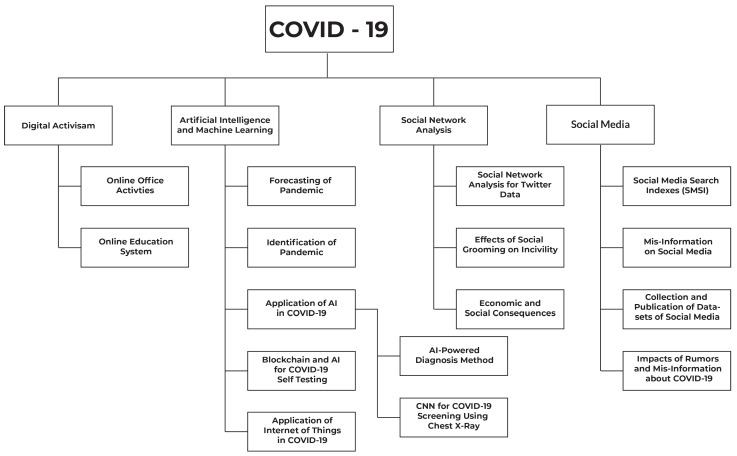
Taxonomy of COVID-19 analysis techniques.

Today, the Internet has become an important part of our lives. Most people spend a lot of time on social media applications, such as Twitter, Instagram, Facebook, Pinterest, and YouTube. COVID-19 has changed the circumstances rapidly. It has shifted the classes and office works from physical to online system (distance learning system). Due to this novel pandemic, almost every activity has shifted over the Internet. Figure 2 shows major components of the taxonomy on which we have conducted our survey.

## 3. Major Components of the COVID-19 Taxonomy

### 3.1. Digital Activism

Digital activism is playing a vital role in the field of digital media. During the novel coronavirus pandemic, the momentum of education has been shifted to digital channels. During this outbreak, governments of various countries have taken the utmost steps to prevent the spreading of COVID-19. Governments have shifted physical education over digital media (digital learning systems). In the section on digital activism, we provide a brief analysis of how COVID-19 has left an impact on education and work in various countries [1].

### 3.2. Artificial Intelligence and Machine Learning (ML)

In the last few years, AI is facilitating human lives. It is playing an important role in almost every field. Most recently, AI is battling very well against COVID-19 in areas such as identification of disease, prevention from disease, and future prediction of disease [22]. There are a lot of applications of AI that are being used to prevent novel coronavirus. In the section on AI, we will provide detail about the various applications of AI that are serving against COVID-19. Furthermore, we will also discuss how AI is used to identify diseases and how it is being used to prevent coronavirus disease. Moreover, we will briefly write about ML and DL techniques, such as chest X-rays screening, imaging data acquisitions, segmentation, and diagnosis for COVID-19 [23].

### 3.3. Social Network Analysis (SNA)

SNA is a method for exploring social structures by using networks and graph theory. In this section of our survey, we will discuss SNA techniques to analyze COVID-19 pandemic data, such as SNA of Twitter data as shown in the taxonomy. Twitter is a well-known social media application and is considered one of the most useful resources for complex social data analysis [24]. In the same section, we will also explain the effects of social grooming on incivility during coronavirus outbreaks. We will discuss mental health and how people show their thoughts on COVID-19 [25].

### 3.4. Social Media

In the current era, social media plays a vital role in everyone’s life. Social media channels are widely used to spread any kind of information anywhere. In short, today’s world is incomplete without social media [26,27]. In the critical time of COVID-19, social media played a positive role as well as a negative role. It has been observed that it is a very common practice to spread misinformation and rumors on social media regarding COVID-19, which ultimately results in an increase in coronavirus and affects human mental health as well [28]. In the section on social media, we will mainly cover three areas (i) the impact of rumors and misinformation regarding COVID-19 on humans, (ii) society’s engagement with social media in the outbreak of COVID-19, and (iii) queries or keywords mostly searched in search engines in the critical time of COVID-19.

We will discuss the aforementioned areas of study briefly in our survey. We will follow our taxonomy of COVID-19 analysis techniques to elaborate our discussion step by step. Section 3 will be based on digital activism and social media, whereas Section 4 and Section 5 will be based on AI and SNA techniques that are used to combat COVID-19.

## 4. Digital Activism and Social Media Conspiracies

During the COVID-19 spread, people shifted their physical activities to the digital world for containment of the novel disease. During this outbreak, online platforms became the prime sources of information for different purposes. Online education started and most of the larger organizations advised their employees to work from home. During this novel outbreak, it has been analyzed that some of the social media users used bots to spread fake news and manipulate data about COVID-19. Furthermore, the researcher worked to collect datasets related to the pandemic and various social media applications and published them for other researchers.

**Figure 3:**
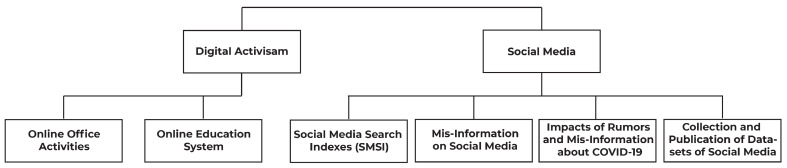
Digital activism and social media analysis techniques.

In this section, we will describe how physical activities shifted to the digital world and how social media applications, such as Twitter, played an important role to spread fake news, as shown in Figure 3.

### 4.1. Digital Activism

Under this section, we will cover how office activities and physical education is shifting towards the digital world and job holders as well as students are facing many issues.

#### 4.1.1. Online Office Activities

The novel virus named COVID-19 compelled many organizations and individuals to operate from home instead of offices. In early 2020, when the virus spread globally, many organizations closed their operations and advised their employees to work from home. However, this is also true that many people were unemployed during the pandemic. Countries recovering from the pandemic are now reopening their offices and some of them have ensured 50% attendance of their employees per day. In [29], 50 world-leading organizations were taken under consideration to look into the actions made during the pandemic. For conducting the research, the content analysis technique was applied to social network posts and web pages to extract 77 activities that were divided into 13 sub-areas and then linked them to a five-level framework that mainly enclosed the functions, such as workforce, leadership, community-related responses, and customers. In the end, it is concluded that organizations were helpful for employees in response strategies and took suitable measurements during the pandemic.

#### 4.1.2. Online Education System

Everyday, we spend a lot of our time on the Internet, in particular on social media apps such as Twitter, Facebook, WhatsApp, and Instagram. However, due to the lockdown situation created by the COVID-19 pandemic, the number of internet users surged to a record high. Most of the office and education activities are shifted from physical to online systems. Today, people are using different apps such as Zoom, Microsoft Teams, and Skype for online meetings. Therefore, it is necessary to conduct research about how the current pandemic affects our businesses, health system, and especially our education system. In [1], a qualitative paradigm along with a descriptive approach was adopted by the author to discuss the effects of COVID-19 on the education system, as well as the negative effects of online learning on the health of humans. The authors discussed that the online learning system is based on applications. These applications are very helpful, however, they are facing some issues, such as signal interference, high data consumption, and so on. Due to online learning, students are facing difficulties in understanding the basic concepts. While some students who belong to backward areas are complaining about the unavailability of the internet and poor signals during online exams. Apart from that, online learning also affects the health of students. Staring at the computer screen for a long time creates eyesight and neck problems, as well as mental problems. In the end, the authors concluded that every social media user must consider the aforementioned problems and try to overcome these problems while using online applications.

### 4.2. Social Media

Here we discuss how people spread fake news over social media platforms, such as Twitter, Facebook, and other apps, during the pandemic. Moreover, we discuss what techniques are applied to extract information from social media platforms and what type of results are compiled from that information.

#### 4.2.1. Social Media Search Indexes (SMSI)

Today, social media is considered one of the main pillars of life. It is also considered the rapid way of spreading any information or news. It is a fact that many websites and social media profiles are designed to pass on fake news and spread misinformation to misguide people on various issues. During the COVID-19 outbreak, many social media users and websites used bots to form and spread conspiracy theories. In [30], the authors performed a comparison between the spreading of links having low credibility information with the articles of the well-known newspaper, New York Times during an outbreak. For this study, two datasets were collected by using different techniques. The first dataset is based on tweets having a set of hashtags and links. Some of the hashtags are linked with the word coronavirus, whereas some hashtags enlighten the various aspects of the pandemic. For their study, the authors only focused on two keywords, #coronavirus and #covid19. The second dataset is based on tweets having links of low credibility of information. The links were fetched, and linked web pages were also extracted. The authors applied various content analysis and network analysis techniques to perform a comparison between tweets having links of incredible information with New York Times articles. The final results showed that the majority of content is posted either by bots or retweets. Social bots played a negative role in promoting tweets with unreliable links.

Twitter is one of the incredible sources to measure public responses. In [31], the author examined Twitter tweets about the COVID-19 pandemic that were posted by Twitter users. The author utilized an ML technique named Latent Dirichlet Allocation (LDA) to identify the famous unigrams and bigrams words. For this purpose, 4 million tweets with 20 COVID-19 related hashtags were analyzed. The final result showed that “lockdown”, “Virus” and “Quarantine” were detected as the most popular unigrams while “stay home”, “social distancing”, and “new cases” were the most common bigram words collected from Twitter data.

#### 4.2.2. Misinformation on Social Media

During the COVID-19 outbreak, many false and manipulated conspiracies are being formed and spread every day on incredible social media applications, such as Twitter, Facebook, and Instagram. Most of the news is based on falsification and has no proof of validity. In [32], the study is divided into two parts and conducted to analyze how many posts were published true and how many were false about COVID-19 on social media. During the COVID-19 outbreak, misinformation was circulated in many forms, such as the idea that a novel virus was created in China to use as a biological weapon, while others claimed that this deadly virus was spread by the United States to get rid of older people who were receiving a pension from the government to decrease the economic burden. The final results of the first part of this study showed that most of the people posted about COVID-19 on social media without deliberation and they had not enough information about COVID-19 to share. The second part of this study concluded that people must think before sharing and validating the accuracy of news of COVID-19 on social media. World leaders across the world are using the Twitter platform to convey important information about the pandemic to their citizens. In [33], content analysis was performed by the authors to examine the tweets posted by the G7 leaders. The data for this study were collected from Twitter with special keywords such as “COVID-19” and “Coronavirus”. The final result of this qualitative research showed that eight out of nine verified accounts of G7 leaders participated in tweets. A total of 166 (82.8%) out of 203 were informative tweets, 14 (6.9%) were political posts and 19 (9.4%) tweets were about encouraging the people.

During the pandemic, much false news was propagated on social media platforms, among them Twitter is considered a leading platform for the spread of misinformation. To deal with this situation the author in [34] performed exploratory research on the data collected from Twitter about COVID-19 misinformation. The author analyzed all the tweets, collected by the fact-checking organizations from January to the middle of July 2020. In the end, it was concluded that 1245 out of 1500 were false tweets while the remaining 276 were false claims about the COVID-19 pandemic. In [35], the author first created a dataset of fake and real news related to the topic of COVID-19. For the proposed dataset, the data were collected from social media platforms and several fact-checking websites. A total of 10,700 posts and articles of fake and real news were collected from social media platforms. Secondly, the author utilized four ML algorithms named logistic regression, support vector machine, gradient boost, and decision tree to classify these posts. The final result showed that the support vector machine delivered good accuracy (93.4%) while the logistic regression delivered (92.75%) accuracy. The authors in [36] used various AI and ML models to help out social media platforms and government agencies by highlighting misinformation on social media. Several ML models, such as Naïve Bayes, LibLinear, LibShortText, and support vector machine, was used during this study. However, the final results reported that LibShortText and Posit were found to be more accurate than others. In [37], the authors used Twitter APIs to collect the data related to the COVID-19 pandemic from 1 March 2020 to 5 June 2020. A total of 85.04 million tweets were collected from 182 countries. After data collection, the authors examined this data to figure out misleading materials and misinformation based on fast-checking sources. To monitor the nature of misinformation, the analysis is displayed on the publicly available dashboard.

In [38], the authors conducted a study for the identification of those hot topics which were posted on Twitter related to the COVID-19 pandemic. A set of tools, such as Twitter API, PostgreSQL database, and Tweepy Python library, were used to collect the words by searching them with predefined special keywords, such as “Corona”, “COVID-19”, and “virus”. Data were collected from 2 February 2020 to 15 March 2020 to conduct the research. Secondly, the authors utilized an ML algorithm named latent Dirichlet allocation to model the obtained articles. Thirdly a sentiment analysis was also performed to obtain the total number of likes, followers, and retweets for each article. The final result of this study showed that out of 2.8 million total tweets, there were 167,073 tweets posted by 160,829 Twitter unique users. A total of 12 COVID-19-related articles were identified and then these articles were categorized according to the origin, source, impact, and steps on how to combat the virus.

#### 4.2.3. Impact of Rumors about COVID-19

Rumors and false conspiracies leave a very negative impact on the community in any event. During the COVID-19 pandemic, rumors, and fake conspiracies have also played a very negative role regarding mortality rate, infected patients, and the origin of COVID-19 disease. Many social media users and websites were involved in the circulation of fake news that ultimately became the cause of people’s depression and fear. In [39], 43.3 million English tweets from the United States were analyzed having the hashtag COVID-19. The main purpose of analyzing tweets was to provide early evidence of how bots are used to spread fake and political conspiracies in the United States. For this study, the authors used the combination of ML and manual validation performed by humans to find out accounts that were involved in illegal activities, such as the automation of tweets and working as bots. However, after this, time series analysis techniques were imposed to extract the aim of humans and bots. The final results of this study showed that the bots were mainly used for the promotion of political conspiracies and fake news whereas human users focused and discussed public health and welfare. In [40], a publicly available dataset of tweets published from 21 January 2020 to 31 March 2020. To extract features such as keywords and past tweets, authors used Twitter API and Tweepy in their study. This dataset consisted of 123 million tweets which contain the hashtag of COVID-19 or even the word COVID-19 in the title. However, around 60% of the tweets in this dataset belongs to the English language. This dataset could also be helpful in future to track the misinformation or rumors about COVID-19. In [41], the authors examined the data collected from social media platforms, government agencies, fact-checking websites, and news agencies to figure out the percentage of rumors, stigma, and misinformation related to the COVID-19 pandemic. The authors performed an analysis using the open-source R statistical package while the content analysis was performed for the data collected from online newspapers and articles. After the analysis, the authors reported 2311 reports of misinformation, rumors, and stigma associated with illness, mortality rate, and the vaccine.

The disaster of COVID-19 has caused significant damage the lives of all kinds of people across the globe. However, the circulation of misinformation on social media platforms misguided individuals and created confusion for them. Therefore, in this context, automated techniques are considered vital tools to identify false arguments in order to avert the propagation of misinformation. In [42], the authors offered a technique based on ML and DL algorithms to check the misinformation related to COVID-19. This study proposed the BERT technique (bidirectional encoder representation from transformers) in combination with deep learning models to find out fake news and conspiracies. In the end, the authors evaluated the BERT technique with five other ML and DL techniques and the results showed that the proposed technique outperformed the rest of the techniques by achieving 99.1% accuracy.

#### 4.2.4. Collection and Publication of Datasets of Social Media

During the COVID-19 outbreak, various researchers worked to collect the datasets of social media applications such as Twitter, Facebook, Instagram and WhatsApp. The main purpose of collecting and publishing these datasets is to provide the facility for other researchers to conduct their research in the future. In [43], a Twitter dataset related to COVID-19 was collected and published for future purposes. The dataset was collected by using Twitter API dated from 22 January 2020 to 13 March 2020. This dataset is based on around 6,468,529 tweets. The main keywords used to search tweets were virus, coronavirus, ncov19, ncov2019, and COVID-19. However, this dataset includes tweets from 66 worldwide languages and the interesting fact was that the English language tweets amount to 63.4% of the total. In addition, the authors intend to use this dataset to examine how information and misinformation were circulated via Twitter during the early stages of the pandemic.

According to rough estimates, there are hundreds of millions of tweets daily on Twitter. It is a well-known medium for publishing information and news. However, it has played an important role in the duration of seasonal diseases such as influenza [44] and even more severe epidemics such as Zika [45], Ebola [46], H1N1 [47], as well as the current pandemic, COVID-19. In [48], the ArCOV-19, named as Arabic COVID-19, dataset of Twitter is presented that is collected from 27 January 2020 to 30 April 2020. This is the first Arabic dataset with 1 million tweets along with propagation networks of the most liked and most retweeted tweets. After this study, ArCOV-19 has been published for other researchers to conduct their research. In [49], a dataset of Arabic tweets is collected by using Twitter API and Tweepy library built in Python from 1 January 2020 to 5 April 2020. This dataset contains around 3,934,610 million tweets. However, the dataset contains tweet features such as id of the tweet, username, hashtags used in the tweet and location from where the tweet is published. The main objective of collecting this dataset is to provide benefits to other researchers and policymakers to explore issues related to COVID-19 in future, such as the prevalence of misinformation, trending hashtags, etc.

In [50], an Instagram dataset related to COVID-19 was presented by the authors to help the research community to study the spread of fake and real news on social media platforms. Instagram APIs were utilized to collect the public data available in the form of posts. While the Instagram Hashtag Engine was employed to gather the data linked with COVID-19 hashtags. The collection process started on 5 January 2020 and continued until 30 March 2020. A total of 5.3 thousand public posts, 392 thousand likes, and 18.5k likes were collected. The authors then organized the dataset into four categories named post contents, such as features, comments contents, and publisher information. Later on, the dataset was made public on GitHub for the researcher community. In [51], a Twitter dataset was proposed by the authors. The authors used Twitter APIs to collect data from 28 January 2020 to 1 July 2020. A total of 63 million COVID-19-related posts from 13 million users were collected. The author then utilized natural language processing and the ML algorithm for similar post-detection and tagging the posts with sentiment valence. In [40], a multilingual Twitter Dataset related to COVID-19 was proposed by the authors that is available on GitHub to help the research community. For this task, the authors utilized Twitter’s streaming API along with the Tweepy library to track the words related to COVID-19 keywords. The data collection process for this dataset was started on 28 January 2020. The authors also utilized Twitter’s search API for the collection of tweets posted in the past. The proposed dataset consisted of a total of 123 million tweets. However, the dataset is being updated regularly. In [52], the authors provided a Twitter dataset named (COVID-19-FAKES) aimed to help the research community to detect misinformation and fake news posted on Twitter. The data collection process for this dataset was started on 4 February 2020 to 10 March 2020. The authors utilized the official websites and Twitter accounts of WHO, UN, UNICEF, and fact-checking websites as a source of valid information to develop a ground-truth database. The authors utilized 13 different ML techniques to annotate the proposed dataset. The proposed dataset is made public on GitHub.

## 5. Artificial Intelligence and Machine Learning (ML)

As the COVID-19 outbreak arose, Artificial Intelligence (AI) also started to play a role in the fight against it. In this section, we will cover several areas regarding AI and DL applications which are helping against COVID-19. In [53,54], the authors presented a detailed comparative analysis of AI-, ML-, and DL-based algorithms used to forecast and identify the epidemic and diagnose the consequences of COVID-19. In [55], the authors proposed a compound model for face mask detection. The proposed technique is a combination of both deep neural and traditional ML algorithms. In the first part of the DL algorithm, ResNet50 was used for high-level feature extraction. While in the second part, traditional ML algorithms named support vector machine, ensemble algorithms, and decision trees were used to detect face masks. Three different datasets were used in this research for the training and testing of the model. One dataset was for training while the other two datasets were used for the purpose of testing. The proposed technique achieved an average of 99.5% accuracy on all three datasets. The research in [20] offers a thorough evaluation of AI and ML as useful methods for tracking contacts, making predictions and forecasting. In [21], the authors discussed a thorough analysis of the current and promising use of AI and big data analytics (BDA) to manage the outbreak based on COVID-19 life cycle stages, such as detection, spread, management, and recovery. The authors also discussed the difficulties that BI in BDA in combat must face.

**Figure 4:**
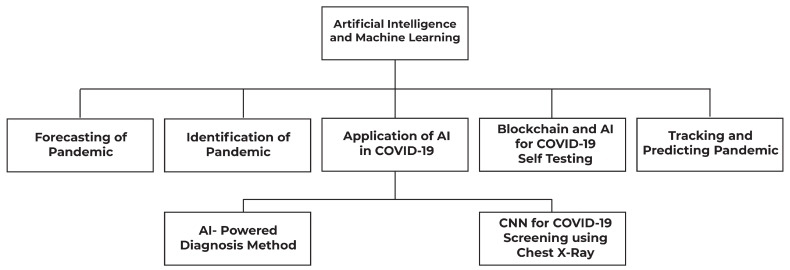
AI- and ML-based COVID-19 analysis techniques.

We will follow Figure 4 to bring forward our discussion in this section.

### 5.1. Forecasting and Identification of Pandemic

AI and ML together are playing an effective role in the detection of various kinds of diseases such as Diabetes, Cardiovascular diseases, and in the detection of various types of cancer. Even now, AI and ML have been proven effective against the COVID-19 outbreak for the identification, forecasting, and diagnosis of novel virus by using various techniques and models. In [56], the authors put their main focus on ML for forecasting. In this area, ML-based techniques have evidenced that these techniques played an important role in improving the power of decision-making on the actions of future work. The authors studied several methods of ML to deal with forecasting issues. This research showed the potential of ML-based architecture to forecast the number of new infections from a novel coronavirus which is currently thought to be a possible risk to humankind. In this study, four main forecasting architectures were studied to predict the alarming factors of COVID-19, such as support vector machine (SVM), least absolute shrinkage and selection operator (LASSO), linear regression (LR), and exponential smoothing (ES). Each model predicted three sorts of information for future purposes, such as the mortality rate, recoveries, and the number of upcoming infected patients. At the end of the research, the authors tested all these models on the dataset and they concluded that exponential smoothing (ES) performed well and near to exactly what they were looking for among all other models. The following other models, linear regression (LR) and least absolute shrinkage and selection operator (LASSO) also performed well to predict the upcoming confirmed cases, recovery and mortality rate of patients affected by COVID-19 while the support vector machine (SVM) performed very poor to make predictions for COVID-19. In [57], the authors put their main focus on ML models and how they are working in real-world situations. For this research, the authors gathered the data from 154 days from the Ministry of Health and Family Welfare of India and analyzed that data regarding the current trend and transmission of patterns of COVID-19 in India. On gathered data, they used supervised ML models which mainly performed an operation of classification of data and regression analysis. In the second phase, they used polynomial regression analysis on the same data. After running both algorithms, they came to know that polynomial regression performed well with 93% accuracy as compared to the support vector machine.

Due to the rapid increase in the number of COVID-19 cases, it is essential to conduct research to understand the rapid growth and spread of the COVID-19 epidemic. Different epidemic models, such as susceptible infection (SI) and susceptible infection recovered (SIR) models, are used for the analysis and prediction of the trend in the growth of the epidemic. However, the output of these models is used to build a good prevention and control mechanism for the epidemic. However, all these traditional models have limitations because they consider all individual COVID-19 patients to have the same infection rate. In [58], the authors proposed a new hybrid AI-based model for the prediction of COVID-19. To deal with the limitations described in the research paper, the authors proposed an ISI (Improved Susceptible–Infected) model to analyze and predict epidemic growth. ISI is an improved multi-parameters epidemic model, applied on many infection periods to perform a deep analysis of COVID-19 transmission. The two main tools are embedded with the ISI model to build a new hybrid model for COVID-19 prediction and to increase people’s awareness and prevention campaigns. One is natural language processing (NLP) and the second one is long short-term memory (LSTM). NLP extracts new information related to epidemic prevention and control measures and encodes it into semantic features. Then these encoded features are passed to LSTM to update the infection rate provided by the improved susceptible–infected (ISI) model.

When the novel coronavirus pandemic came into existence in early 2020, researchers and experts started to predict that there will be waves of cases or a pattern that was seen in other virus pandemics of the last century. The novel virus is continuously transforming itself into different forms. However, as we are writing our survey paper, the fourth wave of COVID-19 named “Delta Virus” is spreading quickly on the earth. Affected cases and death tolls are once again increasing day by day. In [59], the authors proposed an ML-based model for the forecasting of COVID-19 in India. They gathered data from 22 January 2020 to 10 April 2020 using the Kaggle website and performed three different models on the gathered dataset, such as linear regression, vector auto-regression (VAR) and multilayer perceptron (MLP). After imposing the above methods on gathered data, they concluded that the cases will rise with every passing day in India. Moreover, the results also showed that the mortality rate and infected cases will decrease slowly in future. In the end, the authors also concluded that MLP (multilayer perceptron) method had performed well to predict cases in future as compared to LR and VAR. In [60], the study has been organized to observe COVID-19 confirmed cases, deaths, and the number of recovered cases in India only. The dataset was built by collecting data from various states of India that contained different classes. On that data, multi-class classification was performed and applied various analysis techniques, such as linear model support vector machine (SVM), decision tree, random forest, and neural network to predict COVID-19 cases. In the end, it is concluded that the random forest model gives high accuracy among all other models. In [61], the combination of ML and soft computing model is presented to replace SIR (susceptible–infected–recovered) models to predict the COVID-19 outbreak. This study showed that two models of ML, such as MLP (multilayer perceptron) and ANFIS (adapted network-based fuzzy inference systems), have performed well and also stated that due to the complex nature of COVID-19, ML is a very helpful tool to model the time series of the pandemic. In [62], the WHO framework is utilized to evaluate and analyze the various applications of AI that are used during outbreak time. In [63], the author proposed an ML-based algorithm (online) for the data collected through the mobile-based survey to quickly predict the presence of the COVID-19 virus. The goal of this model was to identify infected people in quarantine areas. In the proposed model different parameters, such as name, gender, location, and a list of symptoms along with duration, were used to collect data from the people. After data collection, an online ML algorithm analyzed these data to predict and report the case to the nearest healthcare centre, as well as to the respondent to take health advice.

Due to the dynamic change in the number of COVID-19 cases, it is very difficult to forecast the rise of cases one week or month ahead. In [64], the authors combined different ML techniques in a decomposed manner to forecast the number of COVID-19 cases for the next six days. The authors used Bayesian regression neural network, k-nearest neighbors, support vector regression, and cubist regression along a variational mode decomposition (VMD) technique to predict the rise of cases for the next few days. The proposed decomposed technique showed a high accuracy compared to single and non-decomposed forecasting models. Forecasting will help the administration to take possible steps to tackle the worse situation. In [65], the authors proposed an ML-based medicine discovery pipeline to screen effective FDA-approved drugs and chemical-related to COVID-19. First, the authors collected data about the proteins that interact with COVID-19 then the authors trained a support vector machine model to forecast restrictive activity and use them to sort chemicals and drugs approved by the FDA. In [66], the authors proposed a hybrid ML-based model to predict the rise of the pandemic and mortality rate for Hungary. The authors combined two well-known models named (MLP-ICA) a multi-layered perceptron-imperialist competitive algorithm and (ANFIS) an adaptive network-based fuzzy inference system to develop the proposed model. The authors prepared a time series dataset and then divide the dataset into two parts. One for training and the other for model validation. After the training, the proposed model delivered a good performance and predict the accurate mortality rate. In [67], the authors employed an artificial neural network along with a grey wolf optimizer to predict the COVID-19 cases. The authors collected time-series data from the WHO website for the training and validation of the model. After training, the model showed good accuracy in COVID-19 case prediction.

In [68], the authors utilized the Random Forest Classification algorithm to forecast the COVID-19 alert level. The authors used Google Trends data collected from 202 countries to train the model. The proposed model achieved 100% accuracy for 5 countries out of 202, 0.8133 accuracy for 154 countries out of 202 and 0.7527 accuracy for a total of 202 countries. In [69], the novel AI-based meta-analysis technique was given to forecasting the tendency of COVID-19 over the earth. Before this, various standard techniques were proposed but were inaccurate due to a lack of efficiency and accuracy. In this research, the novel technique was mainly proposed to fill the loophole of previous techniques. The well-known and powerful ML algorithms, such as support vector machine, linear regression, and Naive Bayes, were used on real-time-series data that contain the global record of deaths, active cases, and recovered numbers of patients. In this study, the 20 most affected countries were targeted. In the end, Naive Bayes gave better and more accurate results than the other three ML techniques. In Table 3, we have discussed a few research papers that used AI and ML models along with their best model.

### 5.2. Application of AI in COVID-19

Today, AI is considered a backbone of the healthcare system, as well as other areas of life. There are countless applications of AI in the healthcare system, such as the diagnosis of many diseases, identification of diseases, and drug discovery. During the COVID-19 pandemic, many countries used AI for various purposes, such as AI-based drones for the surveillance of the public and the delivery of drugs to patients. Some developed countries used AI-based models on patients’ chest X-ray images affected by COVID-19 to predict, identify, and diagnose the disease. In [70], the authors used various techniques based on AI and DL, such as Generative Adversarial Networks (GANs), Extreme Learning Machine (ELM), and Long/Short-Term Memory (LSTM). The major aim of the proposed AI-based techniques is to speed up the process of diagnosis and remedy of the COVID-19 illness. In this paper, the authors proposed conceptual AI-based approaches for dealing with COVID-19 issues. The authors added various other techniques to diagnose COVID-19 systems including RNN, GAN, ELM and LSTM for choosing the models of estimation and identification of parameters taking the number of clinical and non-clinical datasets into consideration.

In [71], the authors discussed various techniques based on AI and ML which are used for the prediction, detection, and combat against COVID-19 at different stages. Biosurveillance is one of the disease detection methods that has been used for many years. Natural language processing, ML, and analytics are play an important role in biosurveillance. The Blue Dot Company located in Canada first detected the COVID-19 pandemic in China by using ML algorithms. SEIR (Susceptible–Exposed–Infectious–Recovered) models have been used to make predictions about COVID-19. These models have also been used for other areas, such as how many cases have been reported and how many patients have recovered from the novel disease. In [72], various AI-based techniques have been discussed related to the COVID-19 outbreak. AI and Big Data help to monitor the real-time growth of the pandemic, screen the drugs and chemicals approved by the FDA, prediction of COVID-19 patients and update government agencies and health departments about the current situation.

Due to the rapid increase in the number of COVID-19 patients, hospitals around the world face pressure of limited medical resources, such as beds, ventilators, and medicines. In such a difficult time it is necessary to predict the patient’s discharge time from the hospital to put down their burden. For this purpose, the authors in [73] proposed a statistical model and ML several algorithms. These algorithms are applied to COVID-19 patients’ real-time data to forecast the discharge time of a patient from the hospital. Several ML models were trained but Stagewise GB predicts the accurate discharge time with high accuracy. We will keep discussing the further applications of AI-powered diagnosis and CNN for COVID-19 in two different subsections.

#### 5.2.1. AI-Powered Diagnosis of COVID-19

In this subsection of forecasting and identification of the pandemic, we will further discuss the applications of AI-powered diagnosis that performed well against COVID-19. In [74], the authors proposed a new model, namely COVIDX-Net, for the automatic diagnosis of COVID-19 patients. Due to the unavailability of COVID-19 public datasets, the authors used 50 chest X-ray images for training the COVIDX-Net. Among 50 chest X-ray images, 25 images were positive coronavirus cases. The COVIDX-Net was comprised of a family of seven CNN models, named VGG19 and MobileNet. After training the models, DenseNet201 and VGG19 showed the best performance and accuracy with an accuracy rate of 90%. In [75], a novel technique is proposed to detect COVID-19 based on built-in smartphone sensors. There are various tools and techniques to detect coronavirus, such as clinical analysis of chest CT scan images and testing of blood. Some other devices also exist which detect the virus at its early stages, such as medical detection kits. These devices are costly and take time to install. A proposed AI-empower-based framework is less costly, efficient, and simple to use, even less educated people can also use it on their smartphone devices. This framework is mainly based on AI that reads the sensors of smartphones and signals measurement to forecast the temperature of the severity of pneumonia and forecast the disease results.

Accuracy unboxed a new riddle for many scientists, researchers, and medical staff in the area of detecting COVID-19 cases [76,77]. One thing is confirmed, that humans are unable to detect COVID-19 disease in patients. For this purpose, many techniques based on the combination of AI and DL are proposed. In [78], a DL model based on CNN (convolutional neural networks) has been proposed which mainly aim to improve the accuracy of reported cases, forecast the disease by using a chest X-ray and identifying disease categorization and the structure of abnormalities to uncover hidden patterns. This model has been imposed on the publicly available dataset to examine the accuracy of the model. The final results showed that this model has achieved an accuracy of around 96.3% and a loss of around 0.151 by imposing on the publicly available dataset from various countries of the world. However, the model indicated 74 negative cases and 32 positive cases along with 3 false-positive cases and 1 false–negative case. In [79], the data of 697 patients who lived in Italy were gathered from 25 February 2020 to 9 April 2020. On collected data, initial chest radiographs and various AI techniques were imposed to forecast the number of patients infected by the COVID-19 outbreak. The final results of this study showed that the number of patients will increase with every passing day as well as the mortality rate will also increase gradually.

When the World Health Organization (WHO) declared COVID-19 as a pandemic. Since then, the researchers and scientific community is struggling to obtain information about the transmission mechanism, new diagnoses and how to prevent and treat patients. In [80], an ML model was used to classify the COVID-19 test results using the joint analysis of well-known laboratory tests based on clinical parameters. In this research, the incumbent connection between laboratory parameters and the results generated by COVID-19 tests are evaluated. On the basis of evaluation, there are two classification models developed and they achieved above 96 % accuracy.

#### 5.2.2. CNN for COVID-19 Screening Using Chest X-ray

In this subsection, we will discuss the applications of CNN for COVID-19 screening using a chest X-ray. In [81], the authors focused on the main issue of how the novel coronavirus spoiled the health of humankind. The authors also wrote a note on PCR (polymerize chain reaction) testing. It is a standard diagnostic tool which is being used to identify the presence of antigens instead of the presence of antibodies and the body’s immune responses. The main challenge to PCR testing was that the ratio of false results spreading was high which ultimately opened the path for alternative testing tools. In this study, the main focus was how we can stop the spreading of false results generated by PCR testing. Another issue with PCR is that it is theoretical testing which means that it can be altered at any stage. In this study, the authors studied the CNN (convolution neural network) techniques based on AI and DL. There were four different models of CNN used such as Inception V3, ResNet50, MobileNet, and Xception while conducting this research. These models were mainly trained for cancer prediction disease. This time these models were run for the COVID-19 dataset and they proved that CNN models have the potential to diagnose COVID-19 disease. The results showed that MobileNet performed best among all the following models, such as Inception V3, ResNet50, and Xception. Due to limited health resources and little availability of COVID-19 infected patient information, it is very difficult to hold research on the prediction and prevention of the COVID-19 outbreak. The reverse transcription-polymerase chain reaction (RT-PCR) is a standard test conducted for the detection of COVID-19 patients. However, some research shows that (RT-PCR) also go through high negative false rates. In comparison with (RT-PCR) the radio, logical image approaches, such as computed tomography (CT) and X-rays, have also played an important and unique role in the field of diagnosis and prediction of novel coronavirus patients with the help of AI techniques. CT images are preferred, compared to X-ray images, because of their three-dimensional image view.

In [82], the authors introduced a novel DL technique for COVID-19 which is mainly based on Lung Infection Segmentation that works on CT image slices to identify the infected region. The authors used a Parallel partial decoder to combine high-level features with the help of paralleled connections because lower-level features require maximum computational resources as compared to higher-level features. The parallel partial decoder then generates a global map as a guide map for the reverse attention module. The reverse attention module is used to enhance the representation and to model the boundaries. To enhance the learning ability and overcome the problem of insufficient data, a semi-supervised segmentation framework is proposed by the author based on a random selection propagation strategy. In [83], the authors proposed a deep convolution network, named COVID-Net, for the detection of COVID-19 patients using chest X-ray images. The COVID-Net model is open source and available for researchers and scientists to use for the prediction of COVID-19 cases. The author also proposed an open-source dataset known as COVIDx that consists of 13,975 chest X-ray images collected from five different open-access databases. The proposed COVID-Net model was trained on the new COVIDx dataset and achieved 93.3% test accuracy. As compared to other techniques of DL and ML the proposed COVID-Net has shown good performance.

The purpose of the work presented in [84] is to evaluate the significance of COVID-19 and post-COVID-19 chest CT findings in both states, such as diagnosis and prognosis of infected patients, as well as to investigate how to incorporate CT findings into the emergence of cutting-edge AI tool-based predictive diagnostic techniques. The study of tracking pneumonia through a chest X-ray based on generative adversarial networks (GAN) and deep transfer learning is presented in [85]. However, this study was conducted on small datasets. The datasets were built of 5863 X-ray images and categorized as normal and pneumonia. To evaluate the accuracy and effectiveness of the proposed model, 90% of the images were generated by GAN and only 10% of the images were used for training data. The study used AlexNet, GoogLeNet, Squeeznet, and Resnet18. However, the final results demonstrated that Resnet 18 outperformed all other models in terms of accuracy.

In [86], the authors proposed a novel technique based on a patch-based convolution neural network (CNN). The main objective of proposing this technique is to overcome the hurdle where the deep neural network faces difficulty to collect the CXR dataset. In the first stage, chest X-ray (CXR) images go through the pre-processed process for the normalization of data. Then these data send through a segmentation network where affected lung points can be extracted. Meanwhile, a classification network comes into action and classifies the disease by using patch-by-patch training. In the end, the final results are manipulated based on majority voting and the final decision is made. In [87], the authors introduced a novel efficient chest radio graphic classification (DL-CRC) technique which is mainly based on DL. This technique is proposed for several reasons, such as cost-effectiveness and ease of availability at public health care centres, even in rural areas. The main focus of this novel approach is to differentiate the novel coronavirus pandemic which came into existence in 2019, with a higher accuracy from other normal and abnormal cases, such as flu and pneumonia. To implement this technique, data are collected from four public sources consisting of the poster-anterior chest view of X-ray data, pneumonia and normal cases and then the dataset is prepared. The introduced technique contains two parts, the DARI algorithm and a two-dimensional convolution neural network model. In the end, results clearly show that this model proved itself very accurate as compared to earlier models. The accuracy shown by DL-CRS is 94.61% which is ultimately much higher than old models. In [88], the authors proposed five convolutions neural networks (ResNet50, ResNet101, ResNet152, InceptionV3, and Inception-ResNetV2) for the investigation of COVID-19 patients by utilizing chest X-ray images. All these models were pre-trained. These models were tested on three datasets obtained from different repositories with four distribution classes, i.e., Bacterial pneumonia, normal, healthy, and viral pneumonia. Among all models, the ResNet50 achieves high performance with an average of 99% accuracy rate.

For training deep neural networks we need rich datasets such as ImageNet. By increasing the number of training examples, the performance and accuracy of the model will also increase. As we all know that the coronavirus outbreak is new and collecting a large number of radiographic images will take a lot of time. Therefore, the authors in this paper present a new method to produce synthetic chest X-ray (CXR) images with the help of the Auxiliary Classifier Generative Adversarial Network (ACGAN) model named CovidGAN. In this proposed technique, the author used a convolution neural network for COVID-19 detection and CovidGAN for boosting the training process of CNN for better detection of COVID-19. ACGAN helps to stabilize the training process of the CNN model and also helps in the production of high-quality images. Results show that the accuracy of CNN increased by 85% to 95% with the help of this new AI technique ACGAN [89]. In [90], the authors trained a novel DL model based on convolution neural network InceptionV3 using X-ray images for the detection of COVID-19 patients to reduce the burden on radiologists. For the model training, the author gathered 260 X-ray images from well-known repositories GitHub and Kaggle, out of which 130 were normal X-ray images and 130 were positive COVID-19 patient images. To investigate the efficiency of the model the author used six well-known parameters, such as accuracy, specificity, negative prediction value (NPV), positive prediction value (PPV), sensitivity, and F1 score. In the end, each parameter achieved a 100% score for the detection of COVID-19.

In [91], the authors proposed two classification models, (i) full 3D model and (ii) hybrid 3D model for the prediction of COVID-19 patients. Both of these models were based on the Densenet-121 framework. For the proposed model training, chest images of 1280 patients were collected from different countries. The proposed full 3D model resamples the full cropped chest image to (192 × 192 × 64) pixels as an input while the hybrid 3D model resets the lung image to (1 mm × 1 mm × 5 mm) resolutions and (192 × 192 × 32) multiple 3D samples as an input to predict the COVID-19 final probability. Both of these models achieved an average of 90% accuracy.

In [92], the authors proposed U-net based AI model for the screening of COVID-19 patients. For the training purpose, the author collected CT scan images of 2447 patients from Tongji Hospital (China) among which 1647 were confirmed positive cases while 800 were COVID-19 negative cases. For external validation, the author collected 2120 CT scan images from three different hospitals. The proposed model achieved high accuracy with a sensitivity rate of 0.923 and a specificity rate of 0.851. The author compared the accuracy with the radiological panel and found that the model successfully identified the COVID-19 cases. In this [93], the author trained an AI model based on the transfer learning rate technique due to less availability of chest X-ray images. The author used two open-source X-ray images dataset to train the model. The proposed framework consists of two models. One is InceptionV3, not a fully connected model while the other is the fully connected neural model with three fully connected layers and a global average pooling layer. After training, the proposed technique achieved 100% accuracy. In [94], the authors compared the state-of-the-art feature extraction deep neural network models. For high-level feature extraction, several models such as DenseNet, ResNet, VGGNet and ImageNet, and some others were used. After feature extraction, the obtained data were fed to several ML algorithms to predict COVID-19 positive or negative cases. The proposed framework was trained on an open-source dataset composed of chest X-ray and CT images. Among all models, the DenseNet121 deep neural feature extractor with the Bagging tree classifier gained good performance with 99% accuracy. In Table 4, we have mentioned a few papers along with testing models and their accuracy.

### 5.3. Blockchain and AI for COVID-19 Self-Testing

The mortality rate and number of affected patients are increasing day by day. In addition to these woes, misinformation and reports based on false data regarding novel disease are continuously spread since the first case reported of COVID-19. Today, blockchain is being used with AI and DL to perform novel and innovative functions in every area of life, such as battlefields, online educational systems, and even now in healthcare systems. In [95], authors have introduced the combination of various emerging and powerful technologies to tackle current issues. These techniques are mainly based on Unmanned Aerial Vehicles (UAVs), AI, IoT, Blockchain, and mobile applications. UAV also known as a drone is a technology that belongs to the category of the IoT, and has played a vital role during the pandemic. Today, drones are being used in many countries to ensure social distancing, public announcements of cautions, spray disinfectants and rapid delivery of medical supplies and other essentials to affected patients. In this short time, AI has grown very rapidly and brought advancements in its technology. During the pandemic, AI technologies have worked vastly and helped the relevant authorities in virus modeling, forecasting risk, medical diagnosis, and development of drugs to fight against COVID-19. Other best practices of AI are very useful to stop the circulation of fake news, host identification, and disease monitoring. In [96], a smartphone application (xRCovid)based on ML is proposed that classifies SARS-CoV-2 serological rapid diagnostic test results. Among 11 other COVID-19 RDT techniques, the xRCovid application achieved 99.3 % accuracy. This smartphone application is well-suited for self-testing.

Blockchain is a distributed and immutable transaction ledger. Once information is recorded into the system then it is very difficult or impossible to change, cheat, or hack the system. Today, blockchain is being started to use in various fields even in banking, battlefields, and now in the healthcare system. In [97], blockchain and AI-based technique is proposed for self-testing and tracking the cases of COVID-19. Getting access to accurate diagnoses needs a well-equipped healthcare system. The main aim of this technique is to enable humankind to self-testing because, in most developing countries, people are unable to obtain access to laboratories. Emerging techniques such as Blockchain and AI can be combined with POC (point of care) to enable patients to self-test. This technique based on blockchain and AI is enough to count the number of positive and negative test results and transfer them to relative authorities. This phenomenon will ultimately help to make sure that all positive cases are advised to isolate their selves for monitoring and suitable treatment.

In [98], the authors proposed a DL-based new global model named Capsule network for COVID-19 pattern detection with Blockchain-enabled technology to securely collect patient data from different hospitals without leaking organization privacy. The authors of this paper also proposed a new dataset with 89 COVID-19 subjects. Among 89 subjects, 68 were confirmed COVID-19 patients cases and 21 were negative COVID-19 cases. The proposed method first collected a large number of data from different hospitals to train the model of a decentralized secure Blockchain-enabled architecture for the latest data related to COVID-19 treatments. As compared to previous techniques, the proposed technique collected a huge amount of data securely and trained a good prediction model. In [99], the authors used an emerging technology, blockchain, in combination with AI that empowers the systems to create a general view of forecasting systems that later on becomes the cause of reducing the pandemic risk on the national level. However, a SWOT (Strengths–Weaknesses–Opportunities–Threats) analysis of the blockchain-based model was performed in this paper. Trust, privacy, and immutability features of blockchain were considered as strengths in SWOT analysis, whereas lack of flexibility, operation costs, and storage space were mentioned as weaknesses of blockchain. Greater and better collaboration among the operators of the healthcare system and an increase in the technological awareness features was considered as opportunity whereas lack of expertise and unchangeable transactions was declared as a threat to the blockchain.

The idea of fog computing is introduced to decrease the burden on the cloud server and to provide a fast response to latency-sensitive applications. So fog computing is a good choice for applications that require fast response. To enhance fast processing, AI is integrated into the IoT. The authors of this paper proposed a new architecture based on AI, blockchain, and edge computing to ensure rapid and secure data sharing and processing. AI is used in this architecture for the analysis of data while Blockchain is used to share data securely without leaking privacy during the COVID-19 pandemic [100].

### 5.4. Application of Internet of Things in COVID-19

The Internet of Things (IoT) is a new and emerging technology that refers to a set of links that interconnect anything, anywhere, with any connection, with any device, at any time. IoT is becoming more popular in many sectors and academic subjects, with healthcare being one of the application areas that leverage IoT devices and sensors for surveillance. Scientists have been using a wide variety of tools to deal with this global epidemic since the deadly virus began, including IoT analytics, which has been developed by the founders of IoT analytics. However, measuring, assessing, and diagnosing risks is made simple with the help of AI. In [101], COVID-19 was inspected in terms of IoT technology and AI. Moreover, evaluating networks, implementing IoT technologies, and leveraging IoT industries are reviewed in relation to their use in fighting COVID-19, including early detection, quarantine times, and post-recovery activities. In addition, this research also investigated how IoT monitors and deals with an outbreak at a new stage of healthcare. In this study, the concept of long short-term memory (LSTM) was used with the combination of the RNN model aimed at diagnosis and, in particular, for analyzing the characteristics of cough and breathing acoustics. However, the final findings indicated that the voice test is less accurate than both coughing and respiratory samples.

In the research article [102], the authors introduced a real-time framework based on IoT aimed at detecting and forecasting deadly COVID-19 at its early stages by using symptomatic data and evaluating the nature of the virus. The introduced framework gathered real-time COVID-19 data from network sensors and IoT devices. The framework is made up of four parts, user system, data analytic center, diagnostic system, and cloud system. In this research, five different ML algorithms are evaluated to detect COVID-19 symptoms in real time by using real-time and primary datasets to validate results. However, the authors concluded that the evaluated ML algorithms have shown 95% accuracy which means the proposed framework is feasible to detect and predict COVID-19 suspects at early stages. An IoT-based wearable body sensor architecture is presented in [103] to combat the COVID-19 outbreak. This research also described that with the help of an internal network, wearable body sensors based on IoT can be used to monitor and control patients’ conditions in cities and towns.

A rapidly growing technology, IoT involves sensors and cloud computing as its key components. Such modern technologies use cloud computing to handle big data and carry out relevant tasks [104]. A wide range of electronic devices has started adopting the IoT standard in the last few years, making IoT one of the most remarkable technologies in this connected world. Transportation, logistics, healthcare, retail, supply chain management, industry, and the environment are a few of the domains where IoT applications have originated and been deployed. In recent years, the IoT has emerged as a new field in healthcare and connected cities and devices have provided a lot of help in tracking and monitoring user data. However, the amount of data created by IoT devices has grown exponentially as the number of connected devices has grown. The present COVID-19 epidemic has entailed the use of Healthcare IoT (H-IoT), which can offer an automatic monitoring solution. As a result, IoT data must be examined thoroughly.

An overview of the current developments in AI applications is presented in [105]. In addition, this research also discussed the role of IoT, data acquisition, preprocessing, and analysis. The authors also thoroughly explained several methods of data preprocessing based on conventional techniques and ML and DL algorithms. As a part of the new way of operating, the authors presented all techniques for handling data preprocessing, analysis, and challenges to IoT. The research presented in [106] introduced a concept and design of an innovative smart helmet that identified coronavirus based on thermal images. For real-time monitoring of the screening process, the thermal camera technology is equipped with smart helmets and IoT devices. In addition to facial recognition technology, the device can also display the personal information of an infected person and can automatically take a temperature, allowing it to detect more infected individuals than manual thermal screening can. However, concerning the diagnosis process, the authors claimed that the proposed design of the smart helmet took less time compared to the normal screening process. The authors of [107] discussed how digital technologies such as IoT, AI, and cloud computing, which have a wider range of applications during this unfortunate crisis, can work together to lessen the impact of the outbreak.

In [108], the author made a valuable contribution to the existing literature by pointing out that Internet of Medical Things data sharing allows wearable medical sensor devices to detect real-time changes in patients’ vital physiological parameters. The Web of Science, Scopus, and ProQuest databases were searched using the terms “COVID-19”, “remote patient monitoring systems”, “wearable Internet of Medical Things sensor devices”, and “deep learning-based computer vision algorithms” throughout January 2022 to conduct a quantitative literature review. However, only 165 articles in research that were published between 2019 and 2022 met the eligibility requirements.

The authors of [109], developed a COVID-19 detector based on the IoT that reduces testing costs by utilizing ML techniques for quick and accurate identification of COVID-19 signs in a patient. The k-nearest neighbor classifier produces accuracy values of 0.995 and 0.996 for the training and test sets of data, accordingly. It demonstrates that the model works well on both training and test sets of data without overfitting. However, the model was tested on nine patients; it reported the results of four as positive and five as negative. The check took a maximum of 28 s and a minimum of 20.5 s to complete, which showed the device’s efficiency and suitability for use in environments where many people are anticipated.

As the COVID-19 pandemic spread, social distancing and quarantine became global norms. IoT systems for health monitoring obviate the need for frequent doctor visits and patient–physician consultations. However, a lot of people need regular medical monitoring and observation by medical professionals. In [110], the authors used the IoT to create a smart system for health monitoring that can have the power to track a person’s temperature, heart rate, blood pressure, and oxygen saturation. However, the IoT system will notify the doctor or physician if any changes are detected in the patient’s health based on standard values. It was discovered that the maximum relative error (%r) in the measurements of heart rate, patient body temperature, and SPO2 were, respectively, 2.89%, 3.03%, and 1.05%.

## 6. Social Network Analysis (SNA)

**Figure 5:**
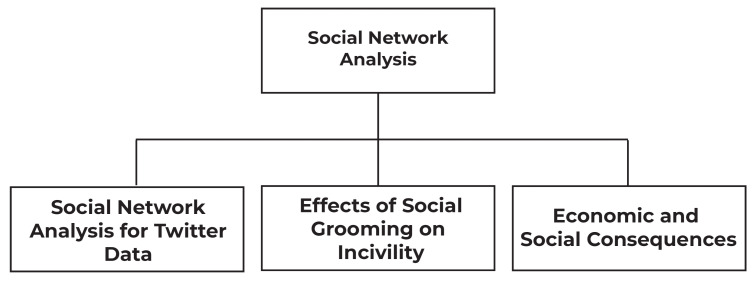
SNA-based COVID-19 analysis techniques.

As the novel virus has spread, the storm of false information and fake conspiracies regarding COVID-19 were trending on social media. We will follow Figure 5 to continue our discussion in this section.

### 6.1. Social Network Analysis for Twitter Data

During any crisis, whether it happens by nature or by humankind, it is observed that people spend more of their time on social media as compared to spending normally. In any crisis, people tend to spend time on two main media platforms, Facebook and Twitter. One of the reasons for these two platforms becoming more active sources is that these social media platforms spread the news very fast compared to news agencies and official channels. During the novel coronavirus pandemic, people posted many posts related to COVID-19 and spread fake news and manipulated results across social media. In [111], the authors worked on the global Twitter dataset named the COV19Tweets Dataset. This dataset has more than 310 million tweets in the English language which were collected from 204 countries and different areas of the globe. These tweets were collected from 20 March 2020 to 17 July 2020. In the collected data, we applied SNA to investigate how many users have included geolocation in their tweets. The results of this study indicated that, out of 310 million tweets, only 141k tweets were made by users which has a “point” location in the metadata. Moreover, the most prominent country among others was the United States, which was in most of the tweets, followed by the United Kingdom, Canada, and India. In the next phase of this study, the COV19Tweets Dataset and geo version of this dataset was further processed for network analysis and sentimental analysis, whereas analysis also confirmed that 12 different communities contributed to mentioning their names while tweeting.

As we are writing our survey, the fourth wave of COVID-19 named “Delta Virus” is spreading rapidly. On the other side, the manipulation and misinformation about the novel virus are also expanding in parallel. The fast expansion of exploited results and false conspiracies has become the cause of producing confusion and fear among people and societies that sometimes lead to deaths. In [112], the research was conducted to build an understanding of the propagators of the COVID-19 conspiracy theory and illustrated the strategies to combat such fake conspiracies. The authors performed their research specifically in the United Kingdom. They used two techniques, such as content analysis and SNA, which were combined and applied to Twitter data for one week from 27 March 2020 to 4 April 2020. At this time, the hashtag #5GCoronavirus was trending in the United Kingdom. SNA figured out the two largest networks based on broadcast and isolated groups. The analysis also revealed that the relevant authorities completely failed to prevent the spreading of conspiracies related to COVID-19. In addition to this, the analysis also confirmed that the websites and YouTube videos with fake news and misinformation were the most shared by users on social media.

In [24], SNA was employed on 2864 users of the most popular social application Twitter and 2775 communications of Twitter. This analysis was only applied to Twitter users who were living in the United States. In the end, the results concluded a few meaningful findings. Firstly, the former president of the United States Barack Obama served an important role in social media, having low compared centrality concerning their followers. On the other hand, Donald Trump, also a former president of the United States, had the strongest impact on social networks. Secondly, this study demonstrated that many other well-known organizations located in other countries such as Nigeria and the United Kingdom acted as important players in the field of communication networks.

In [113], the main objective of the study is to analyze the diffusion pattern and the information spread on various online platforms of the COVID-19 virus. However, in this study, data from only the Twitter application is analyzed. To capture virus diffusion patterns, a SIR model (named as SIRsim) was created which was mainly based on empirically validated transmission methods of COVID-19 and then these patterns were compared with actual cases of the virus for specific durations. On the other hand, to confirm the diffusion patterns of information which was shared online about COVID-19, three cascades (named as INFOcas) were created based mainly on Twitter retweets, reply tweets, and quote tweets that had the keyword of COVID-19. In [114], the updated deep neural network has been suggested for the identification of fake news and misleading conspiracies. In this study, the DL techniques are the Modified-LSTM and the Modified GRU (one to three layers each). To conduct this study, a large dataset of tweets concerning COVID-19 was collected and classified uncertain data into true and false categories. The six ML techniques, named logistic regression, naive Bayes, decision tree, support vector machine, random forest, and k-nearest neighbors, have been applied to four different benchmark datasets. The parameters of six deep learning techniques are optimized by using a Keras-tuner. In the end, the acquired results with the given framework uncover the higher accuracy concerning detecting tweets containing fake and non-fake information about COVID-19.

During the crisis of novel coronavirus-19, a variety of different fake news, theories, and altered results infected patients of COVID-19 were spread out on various online social media applications. This activity of work is described by a new term that is “Infodemic”. In [115], the study was conducted to analyze the properties and behavior of the infodemic on Twitter. A dataset consisting of 9958 tweets, posted by people who were living in South Korea, were gathered and almost 14,211 relationships were studied by applying SNA methods. All these tweets, reply tweets, and quote tweets contained the keyword ’infodemic’ which created the relationships between tweets.

In [116], the research was conducted to analyze the conversation topics of online users and conversations between users on Twitter regarding the “Liberate” protest movement against social distancing government instruction at the start of the COVID-19 outbreak. To achieve this work, ML, SNA, content analysis and interdisciplinary techniques in Big Data were used to detect the conversation themes, characterizing the communicative behavior and network structure of supporters and non-supporters. The final results demonstrated a significant difference between the supporters and non-supporters of the protest. The results suggested that those in favor of protest are more consistent, shared and unitary. Furthermore, the network of supporters is well positioned to establish a clear and meaningful message that the other users can easily understand.

At the end of January 2020, the WHO declared a public health emergency due to a significant increase in the number of patients infected with COVID-19 and declared the novel type of coronavirus as a pandemic on 11 March 2020 [117,118]. The WHO also suggested drawing a pandemic plan to eliminate this novel disease. Today, newspapers and media are playing a vital role and become the authentic source of information for folks during any pandemic crisis [119]. One of the popular social applications, Twitter, played an important role in engaging people with the latest updates regarding mortality rates, infected patients, and recovered patients during the COVID-19 outbreak.

In [120], the tweets of two main Spanish newspapers, El País and El Mundo, were targeted to analyze and compare the news of both platforms during COVID-19 using SNA techniques. In this study, eight news frames were identified for each Twitter account newspaper. However, the division of the whole duration of the pandemic was separated into three periods, pre-crisis, the lockdown period, and the recovery period. The final results concluded that El País strongly focused on the news regarding public health and real-time alarming information, whereas El Mundo focused on the state of alarm and confinement-related information in the first two periods of the pandemic. In the third period, named the recovery period, El País changed its perspective and put its main focus on political news, while no change was observed in the results of El Mundo during the pandemic. In Table 5, we have mentioned some research papers along with dataset size, API, and their SNA techniques.

### 6.2. Effects of Social Grooming on Incivility

The COVID-19 pandemic affects every aspect of human life, whether it is economic, educational, physical, or whether it is the mental aspects of our life. It also badly affects the education system around the globe. Educational activities have been shifted from a physical system to an online system. The online education system has created black holes for the business models of the universities as well as creating problems for the students. Students with poor financial and social backgrounds have been badly affected by this pandemic. In [26], the authors proposed SNA for creating good support for students belonging to different backgrounds. The authors proposed different recommendations for the university staff, as well as for the students that need to be considered while developing effective support. The authors outlined the strength of social networks in understanding the support that students belonging to different socio-economic backgrounds require. The authors first outlined the role of their professional network and capital while developing effective student support. It is necessary to develop such a support system that provides benefits to students with middle-class backgrounds. It is very necessary to investigate the inequalities in our education system as, e.g., some students have very limited access to technological tools and they cannot afford the necessary devices that help them to access the online support provided by the universities. It is necessary to engage students with small, effective social networks to receive the necessary support they need.

In [25], the authors performed a deep analysis with the help of a modern computer-assisted analyzer to discover which social grooming factor minimizes user’s incivility on social media when commenting and posting about the COVID-19 pandemic. COVID-19 has affected all countries around the globe with a high mortality rate amongst elderly people. Due to this tragic situation, people are expressing anger and hostility on social media, targeting race, religion, and nationality. The authors performed a semantic network analysis to extract how people express their feelings. The author used an API-based data crawling software named Mozdeh to collect COVID-19 relevant tweets from Twitter and then performed a refining process on these tweets to eliminate irrelevant data. To extract bad words from the collected Twitter tweets, Linguistic Inquiry and Word Count (LIWC) software is used. LIWC is a text analysis software. Network size, the number of positive responses, and the number of posts are social grooming factors used for analysis. The results showed that a bigger network size does not lead to incivility while the number of positive responses and the number of posts are related to the degree of incivility.

COVID-19 caused significant damage to the economies of developed countries, as well as middle- and lower-income countries across the world due to its direct impact on the livelihoods and health of the people living in those countries. In [121], statistical and SNA techniques were employed on the data of 1212 patients affected by COVID-19 in Henan of China. This study calculated that the average, mode, and median periods of incubation are 7.4, 4, and 7 days, respectively. This study also concluded that the incubation interval of 92% of affected patients was less than or equal to 14 days. Moreover, the study also concluded that the workers and students of schools and colleges are at high risk. Due to the rapid increase in the positive cases of COVID-19, it is necessary to pay attention to pandemic risk estimation. The authors in this paper used a scientific approach named network analysis to figure out the direct visualization of COVID-19 pandemic risk. Based on confirmed cases the author shows the degree of connectedness among different geographic regions. Network analysis is a good technique to estimate the COVID-19 pandemic risk [123]. In [124], the location network analysis (LNA) technique based on ML was considered, an approach that aimed to predict an accurate new number of possible outbreaks.

### 6.3. Economics and Social Consequences

The novel coronavirus has disturbed every area of life and caused several kinds of issues to develop, including several forms of anxieties, travelling outages, distress, and individual avoidance. Some people think that the warnings surrounding COVID-19 is, itself, anxiety inducing. The anxiety relating to COVID-19 is more than a concern about infection. In [122], the authors performed a network analysis to search how these things are interrelated. To conduct this study, a sample of 3075 adults living in America and Canada were collected through an online survey relating to topics such as avoidance, self-protective behavior, COVID-19-related worry, among others. After collecting data through an online survey, the results were categorized into three main hubs, such as worries about COVID-19, a belief that the threat of COVID-19 is exaggerated, and self-protective behavior. Then, the network analysis techniques were used to investigate the link between COVID-19 and the data collected.

In [125], the study was conducted to investigate the substance use and consumption of alcohol during the COVID-19 pandemic. The data of 3075 adults living in the United States and Canada were collected through an online survey form. The sample was mainly based on age, geographic region of the participants within each country, gender, and ethnicity. In the end, SNA methods were run that collected information from various people and results have shown that, during COVID-19, the consumption of alcohol and drug increased to 23% and 16%, respectively. Moreover, network analyses also indicated that stress symptoms and disregard symptoms regarding COVID-19 are negatively correlated. This simply means that they both have a positive connection with alcohol and drug abuse.

In [126], the author used SNA and its tools to monitor and control the COVID-19 outbreak in the Indian state of Karnataka. The author collected publicly available data from 1147 patients of different ages and genders. Two SNA tools, named Gephi and Cytoscape, were used to show SNA graphs and to show the edges and nodes. The attribute node was determined for positive cases and attribute edges were determined so that the main source could target patient-directed links. The conducted study shows that middle-aged and young men were mostly affected by COVID-19 positive cases, while elderly people suffered from a high death rate. According to the author, SNA is a good tool to detect COVID-19 hotspots, helping government bodies to take precautionary measures in those areas. In [127], the authors conducted research based on SNA to assess the Indonesian Government Administration’s responses to the COVID-19 pandemic. The author used the Gephi tool to produce SNA graphs. The data were collected from the magazine Tempo. There were 150 actors that represent nodes and a total of 180 connections that represent edges. In the end, the conducted study showed that there was a lack of coordination between government agencies. They demonstrated that the head of the COVID-19 task force paid very little attention to the pandemic, and the study also noticed that there was a lack of medical equipment and significant violations of social distancing rules.

As we already mentioned above, due to the COVID-19 outbreak, teaching shifted from a face-to-face system to an online education system. In [128], the authors performed questionnaire surveys and web crawlers to collect user experience data about the online education system for quantitative analysis. Furthermore, to forecast the user prediction, the author builds a back propagation neural network. The authors select several online teaching platforms to find primary, middle, high school, and university students to collect data. After collecting the data, the refining process was performed to remove irrelevant data, and then the data were entered into SPSS statistical software to perform the analysis. In the end, the author outlined several online platforms based on good quality. In addition, it was found that the availability of a good platform has a positive impact on user satisfaction. Furthermore, the BP neural network that was designed for future prediction enabled 77.5% effective accuracy.

It is very important to understand the impact of COVID-19 during the early stages of the pandemic. To highlight the current COVID-19 hotspots and outline future directions the author performed a bibliometric analysis to obtain COVID-19 approximate data. For this purpose, the data are gathered from the Scopus inventory, and to analyze the data the author used bibliometric indices, such as document type, country, journal name, and citation patterns. According to the bibliometric analysis, it is indicated that 19,044 publications were published on Scopus during the early outbreak of COVID-19. Among which, 9140 (48.0%) were articles, 1797 (9.4%) were review documents, 1728 (9.1%) were notes, and 4192 (22.0%) were letters. The USA published the highest number of publications, China published the second greatest amount, while Italy published the third most [129].

According to WHO facts and figures, the United States is the country with the highest percentage of deaths and infected patients caused by COVID-19. In [69], the network analysis has been performed on reports of daily confirmed cases of COVID-19 to investigate the connectedness of the network. Moreover, in this study, the authors have targeted five states in the US where the percentage of COVID-19 cases is very high. A scale-free and efficient tool is proposed by the authors, which is mainly used to detect and monitor the pandemic, as well as the connectedness of states to demonstrate some helpful information for law agencies to take measurable actions at the right time.

In [130], the study mainly focused on analyzing the content uploaded to Twitter concerning the keyword ‘mask’ during the pandemic. The data of tweets regarding the keyword ’mask’ from Twitter were gathered from 27 June 2020 to 4 July 2020. The total number of tweets considered for the study was 452,430. From the gathered data, SNA techniques were imposed to analyze the results. The final results showed that the keyword ‘mask’, during the outbreak, was most commonly used to encourage people to wear a mask.

## 7. Discussion and Future Directions

AI- and ML-based techniques and models have found significant potential in the global war to combat COVID-19 and have been used for a variety of different applications, such as identification, forecasting, and diagnoses of the disease by analyzing COVID-19 datasets. Apart from the positive consequences, here we highlight a few of the key lessons derived from this research and also give some recommendations based on learnt lessons for the research communities. In addition, we would like to mention a few problems and challenges that need to be addressed in future.

### 7.1. Findings of the Research

The COVID-19 pandemic was identified in 2019 and has claimed millions of lives within two years [2]. Due to the novel disease, many approaches and methods have been applied to try and restrict the spreading of COVID-19, and most of the techniques were based on COVID-19 data analysis, such as lockdowns, social distancing, wearing masks, hand cleaning with sanitizer, among others. Still, most countries are striving to control the novel virus.

The lack of limited and standard datasets has proven one of the critical barriers the maturation of AI and ML applications as trusted tools to fight against the virus. To accurately predict outbreaks of the disease, various AI and ML algorithms have been introduced, but they have all been tested on different sizes of datasets from different regions. For example, the algorithms in [131,132] are tested on different sizes of datasets and result in accuracy of around 82.9% and 98.27%, respectively. However, based on the results of both algorithms, we cannot say which algorithm is better in detecting the virus due to the fact that the algorithms have been tested on different datasets with different numbers of samples. Furthermore, at the beginning of the pandemic, data on COVID-19 were not collected or organized in a structured and coherent manner, and therefore the processing of these data is also limited, which yields limited results. On the other hand, some researchers have collected scattered data from the Internet and have unified them in order to create their own datasets without any credibility. Furthermore, many of the datasets were inauthentic and resulted in the faulty data processing. Some of the datasets contain irrelevant or mixed images of the chest X-rays of minors and elderly people on which the models were trained that were not applicable in real life.

To tackle these challenges, governments, credible authorities, health organizations, and big organizations need to work together to create high-quality, larger [133], and benchmark datasets. Furthermore, researchers need to train their AI and ML models on large datasets with real data in order to obtain better insights in terms of accuracy and specificity. Table 6 shows AI and ML techniques and models that were used in the literature related to COVID-19 data analysis.

COVID-19 has disturbed the lives of all people across the Earth. After the initial spread of COVID-19, many waves of the pandemic have been witnessed in many parts of the world. Although digital technologies have been a huge help in the fight against COVID-19, they have also laid the groundwork for weaknesses that can be exploited in terms of negative social behavior. The amount of manipulated information, misinformation about the novel virus, and racial hatred [134] has also expanded and been published. The fast expansion of manipulated results and false conspiracies has caused an increase in confusion and fear among people, sometimes leading to deaths. However, the detection of fake news is already an area of significant interest in the social and data science community.

There are also future challenges related to the theme of our article, including (i) using natural language processing (NLP) to analyze public opinion regarding COVID-19 policies from social media, (ii) using NLP to infer scientific findings of COVID-19 from scholarly articles, (iii) identifying social and health risk factors for COVID-19 infections (obesity, air pollution, etc.), (iv) predicting COVID-19 deaths and infection numbers at a city and county level, (v) considering social and ethical issues when analyzing patient data, and (vi) determining which subgroups are at a higher risk of infection from incumbent cases and trends. Other than these, there are a variety of challenges and competitions being held on Kaggle and other sites to address issues relevant to open-source COVID-19 datasets.

### 7.2. Future Work

In this survey, the following recommendations are made for future work. Firstly, as we witnessed, many social media users spread fake news and incorrect death numbers in relation to the novel coronavirus. To combat fake news and misinformation, we should have to train strong computerized systems and empower them enough to detect fake news using AI, ML, and DL, which obtain the keywords of the post and then double-check the content of the post. If the model finds a post that has manipulated or false information then the handler should be punished and the account should be blocked permanently or for a limited time. Furthermore, during the pandemic, people heavily rely on technologies such as social media, online shopping, entertainment, and banking, and it is necessary to address and mitigate their misuse.

The COVID-19 outbreak disturbed the typical systems of the educational system, as well as office work activities. In the period of the COVID-19 pandemic, we have seen that office work and education almost entirely shifted from physical to online, which caused unemployment for thousands of people worldwide. Although, it is a pandemic which affected every individual in some aspect, in many countries the swtich to an online education system has not proved as successful. During this outbreak, in many countries, and especially in less developed countries, the experiment of learning via online platforms was not successful; however, the COVID-19 pandemic has also unlocked new ways to utilize new technologies, such as AI-, ML-, and DL-based models, as well as IoT devices to improve education systems and online workplaces. In future, we have the opportunity to develop a strong online education system, such as AI- and ML-based personalized learning systems; AI- and IoT-based virtual classrooms; the development of chat-bots and assistants to help students and teachers by answering questions and providing opportunities for discussion; AI-based automated grading systems and feedback, to automate the grading systems and save teacher’s time; and remote invigilation to monitor activities of students during exams. In contrast, the presence of students/teachers and relevant infrastructures should also be assured by the development of AI-based early intervention systems and support in order to monitor students’ and teachers’ attendance and to provide help.

As we have seen during the pandemic, the lack of limited and standard datasets has proven one of the critical barriers for the maturation of AI and ML applications as becoming trusted equipment to fight against the virus. AI and ML models need to be trained on huge amounts of real-time data; however, IoT devices can also be further improved by using the latest sensing and AI techniques.

## 8. Conclusions

The rapidly spreading novel coronavirus has affected everyone in some way. COVID-19 rapidly spread worldwide within weeks. The whole world is still striving to eliminate this deadly pandemic that claimed millions of deaths. In this survey, we have presented a state-of-the-art survey on almost every aspect related to COVID-19 data analysis. In our survey paper, firstly, we discussed the novel virus and the background of various pandemics that occurred in the previous century; then we discussed digital activism and the key role of social media. Furthermore, we have addressed how social media platforms have played a role in spreading fake news and manipulated results regarding COVID-19, as well as the shift of physical office work and education to online spaces. We have also reviewed AI-, ML- and IoT-based techniques that played an important role in combatting COVID-19 in many ways. Many countries used AI-based drones to disperse crowds in order to ensure social distancing, for supplying medicines to patients, and to spray antiseptic into the air to kill the virus. We also have described the working of some techniques based on AI to detect, predict, and diagnose COVID-19 virus infection. Furthermore, we have also reviewed the techniques of SNA that were used to find the various results, such as how many times the keyword ’COVID-19’ or ’outbreak’ was used on social media in a specific country. In the end, we have discussed challenges that need to be overcome and addressed, and we also recommend some possible solutions for the success of AI, ML, and IoT techniques and models. Although this paper has provided an in-depth analysis of published studies covering a wide range of research areas related to COVID-19, researchers and scientists can take advantage of this detailed survey paper to design and develop future AI- and ML-based models and techniques. This survey paper contains a detailed study related to SNA and digital activism which can help researchers to find future paths. However, it does offer a realistic perspective and a fair comparison of the studies that have been conducted in this field over the past two years, which can help other researchers to determine where to focus their future efforts.

## Figures and Tables

**Table 1:** Comparison of different pandemics.

**Disease**	Spanish Flu	Asian Flu	Hong Kong Flu	SARS	COVID-19
**Causative Agent**	H1N1	H2N2	H3N2	SARS-CoV	SARS-CoV-2
**Years(s)**	1918–1919	1957–1958	1968–1969	2002–2004	2019–Present
**Death Numbers**	Approx. 50 Million	Approx. 1.1 Million	Approx. 1 Million	774	6.59 M (till 11-02-2022)
**Classification**	Pandemic	Pandemic	Pandemic	Outbreak	Pandemic

**Table 2:** Comparison with Existing Research.

Ref. No.	Artificial Intelligence	Machine Learning	Deep Learning	Internet of Things	Blockchain	Social Network Analysis	Impact of Social Media
[8]	✓	×	×	×	×	×	×
[9]	✓	✓	×	×	×	×	×
[10]	✓	×	×	✓	×	×	×
[11]	×	×	×	✓	×	×	×
[12]	✓	✓	×	×	×	×	×
[13]	×	×	×	×	✓	×	×
[14]	✓	✓	✓	×	×	×	×
[15]	×	×	✓	×	×	×	×
[16]	×	×	×	✓	×	×	×
[17]	×	✓	×	×	×	×	✓
[18]	×	×	×	×	×	✓	×
[19]	×	×	×	×	×	×	✓
[20]	✓	✓	×	×	×	×	×
[21]	✓	×	×	×	×	×	×
Our Survey	✓	✓	✓	✓	✓	✓	✓

**Table 3:** Forecasting and identification of pandemic.

Ref. No.	Technique	Models	Outperforming Model
[56]	Machine Learning	SVM, LESSO, LR, ES	ES (exponential smoothing)
[57]	Machine Learning	LR and PR	PR (polynomial regression)
[58]	Hybrid AI-based	ISI with NLP and LSTM	-
[59]	Machine Learning	LR, VAR, MLP	MLP (multi-linear perceptron)
[60]	Machine Learning	Linear Model, SVM, RF, DT, NN	RF (random forest)
[66]	Hybrid	MLP-ICA, ANFIS	-
[69]	Hybrid	SVM, LR, NB	NB (Naive Bayes)

**Table 4:** Application of AI to combat COVID-19.

Ref. No.	Model	Dataset Size	Accuracy
[74]	CovidX-Net Family	50 Chest X-ray Images	90%
[78]	CNN	106 Chest X-ray Images	96.3%
[80]	ML Classification	-	96%
[83]	COVID-Net	13,975 X-ray Images	93.3%
[87]	DL-CRC	-	94.61%
[88]	ResNet Family	3 different datasets	99%
[90]	InceptionV3	260 X-ray Images	100%
[91]	Full 3D, Hybrid 3D	1280 Patients Images	90%
[92]	U-Net AI model	2447 Images and 2120 Images Datasets	92.3%

**Table 5:** Social network analysis research work.

Ref. No.	Location	Database API	Database Size/Collection	SNA Technique
[25]	Global	Twitter	-	Semantic Network Analysis
[111]	Global	Twitter	310 Million Tweets	Social Network Analysis, Sentiment Analysis
[112]	United Kingdom	Twitter	27 March 2020–4 April 2020	Content Analysis, Social Network Analysis
[115]	South Korea	Twitter	9958 Tweets	Social Network Analysis
[121]	China	-	1212 Patients	Statistical Analysis, Social Network Analysis
[122]	USA, Canada	-	3075 People	Network Analysis

**Table 6:** COVID-19 analysis techniques in related work.

Ref. No.	Purpose	Technique
[55]	Forecasting of COVID-19	Combination of machine learning techniques
[56]	Forecasting and identification of pandemic	Exponential smoothing
[57]	Analysis and prediction of COVID-19 in India	Polynomial regression
[58]	Prediction of COVID-19	Hybrid-AI-based model
[60]	Detection of CoVID-19 active, deaths and recover cases in India	Linear model, SVM, decision tree, random forest and neural network
[65]	Prediction and reporting of COVID-19 cases	Machnie learning algorithms
[66]	Prediction of COVID-19 outbreak	MLP and ANFIS
[68]	Forecasting of COVID-19 patients	Random forest algorithm
[70]	Diagnosis of COVID-19	AI-based models (RNN, GAN, LSTM, ELM)
[74]	COVID-19 patients diagnosis	COVIDXNet architecture
[75]	Detection of COVID-19 and forecasting of disease	AI-based smartphone sensor
[80]	Classification of the COVID-19 test results using joint analysis	Developed two classification models based on ML
[81]	Improving PCR test results	CNN model (Mobile Net)
[82]	Lungs infection segmentation	Deep learning techniques
[86]	Classification of disease	Patch-based CNN
[87]	Classification of COVID-19	Deep learning Chest Radiographic Classifier (DL-CRC)
[88]	Classification of COVID-19	ResNet-50
[89]	Detection of COVID-19	CNN-ACGAN
[90]	Detection of COVID-19	CNN model (InceptionV3)
[91]	Prediction of COVID-19 patients	Full 3D model and hybrid 3D model
[92]	Screening of COVID-19 patients	U-net based model
[94]	Prediction of COVID-19	DenseNet121, deep learning neural feature extractor with bagging tree classifier
[96]	Developed self-testing smartphone application (xRCovid)	ML-classifier-based application to classify serological RDT results
[114]	Identification of fake and not-fake tweets on Twitter	Logistic regression, naive Bayes, decision tree, SVM, random forest, K-nearest neighbors

## Data Availability

Not applicable.
